# Supplementary figures and images for: Analysis of the NK2 homeobox gene ceh-24 reveals sublateral motor neuron control of left-right turning during sleep
Source: eLife. 2017 Feb 28;6:e24846. doi: 10.7554/eLife.24846 (PMC5384828; doi:10.7554/eLife.24846)

A

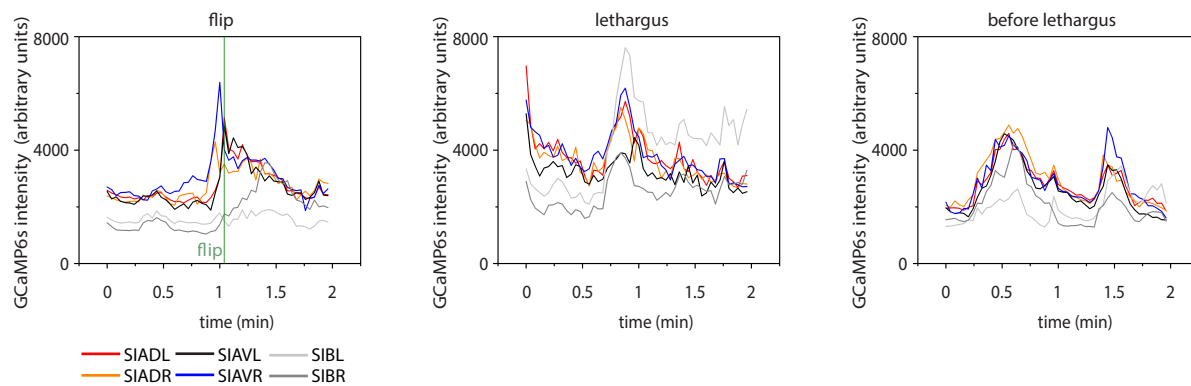

B

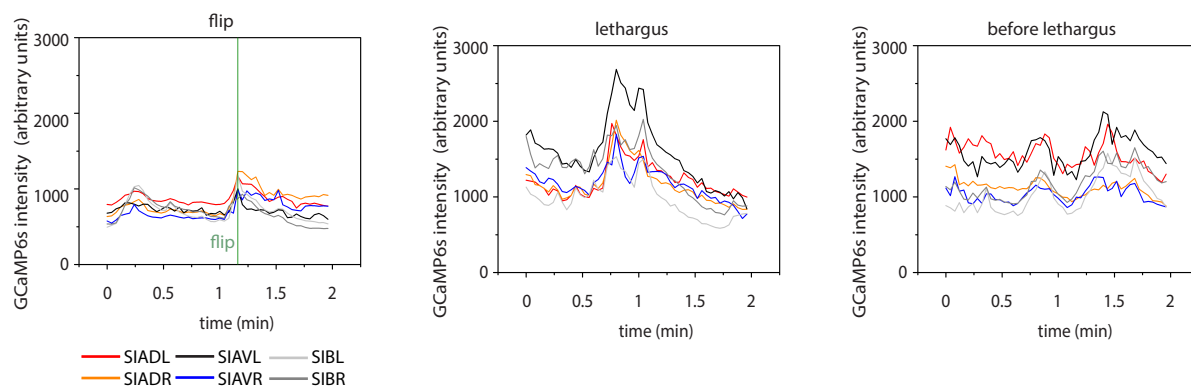

C

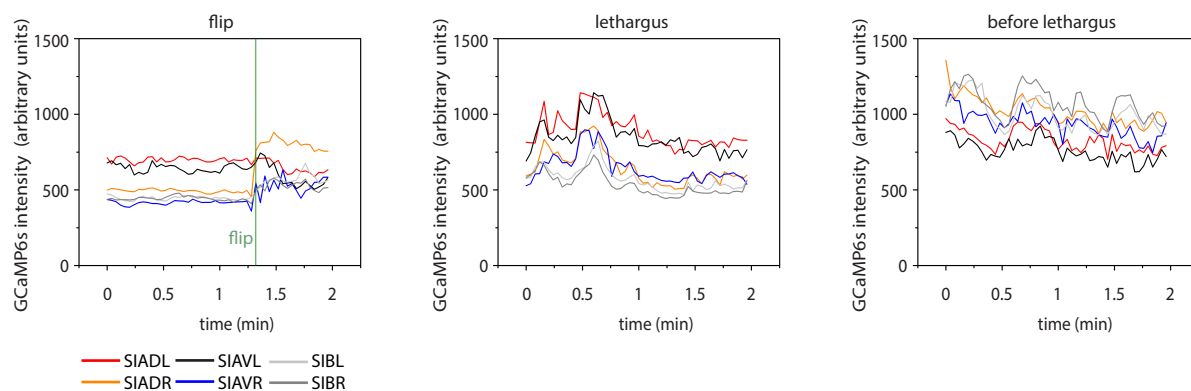

D

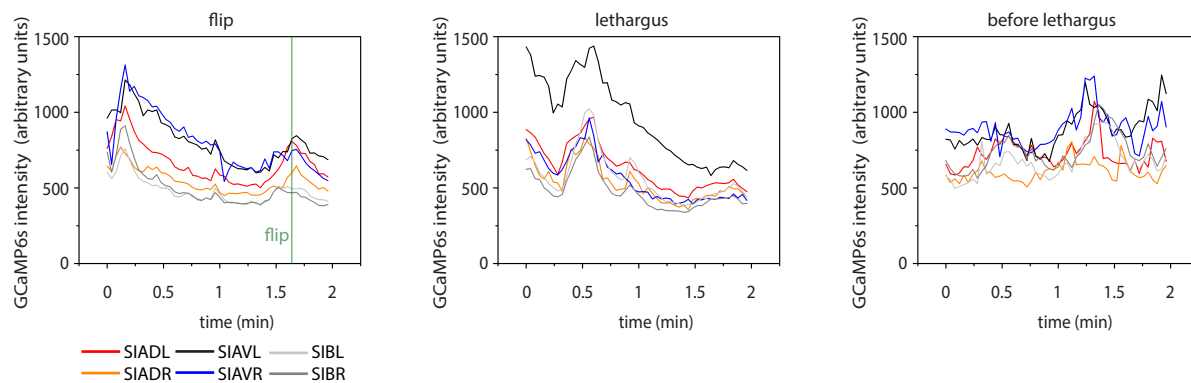

# Supplement 6, Schwarz and Bringmann

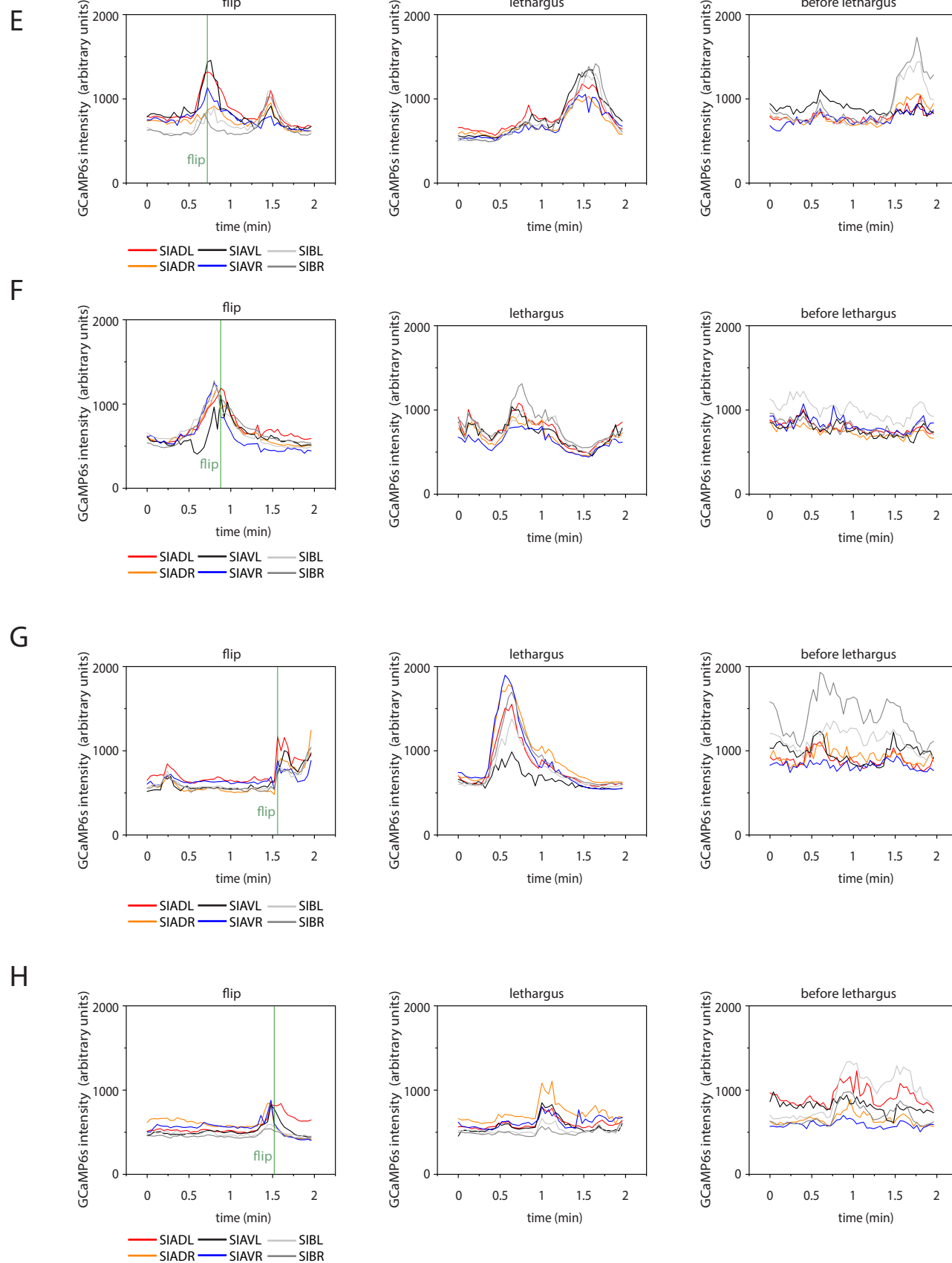

Supplement: Figure 5—source data 1. — The SIAs activate with different transient strengths relative to each other and variably during and outside of lethargus: Individual SIA activation patterns during wake were unequal regarding their transient strengths and highly variable between activation transients. Each row (A–H) contains three example traces from the same individual worm, one example is shown for a flip, one for a trace containing transients during lethargus without flipping and one for a trace outside of lethargus. DOI: http://dx.doi.org/10.7554/eLife.24846.011 [file elife-24846-fig5-data1.pdf]
